# XPS study and electronic structure of non-doped and Cr^+^ ion implanted CuO thin films

**DOI:** 10.1038/s41598-025-08421-4

**Published:** 2025-07-12

**Authors:** Katarzyna Ungeheuer, Amelia E. Bocirnea, Konstanty W. Marszalek, Waldemar Tokarz, Denis A. Pikulski, Zbigniew Kąkol, Aurelian C. Galca

**Affiliations:** 1 Faculty of Computer Science, Electronics and Telecommunications, AGH University of Krakow, 30 Mickiewicza Ave., 30-059 Krakow, Poland; 2 Laboratory of Surface and Interface Science, National Institute of Materials Physics, Atomistilor 405A, 077125 Magurele, Ilfov Romania; 3 Advanced Diagnostic Equipment Sp. z o.o., 79 Tetmajera St., 31-352 Krakow, Poland; 4 Faculty of Physics and Applied Computer Science, AGH University of Krakow, 30 Mickiewicza Ave., 30-059 Krakow, Poland; 5 Faculty of Metals Engineering and Industrial Computer Science, AGH University of Krakow, 30 Mickiewicza Ave., 30-059 Krakow, Poland; 6 Laboratory of Complex Heterostructures and Multifunctional Materials (HeCoMat), National Institute of Materials Physics, Atomistilor 405A, 077125 Magurele, Romania; 7 International Centre for Advanced Training and Research in Physics, Atomistilor 409, 077125 Magurele, Ilfov Romania

**Keywords:** DFT, Ion implantation, Thin films, Electronic structure, Copper oxide CuO, XPS, Condensed-matter physics, Surfaces, interfaces and thin films

## Abstract

CuO is a p-type semiconductor that can be found useful in various applications, sensing, photocatalysis or photovoltaics. Better material performance can be achieved by doping. In our study the doping was done using Cr ions and implantation method. Thin film samples were characterised with X-ray photoelectron spectroscopy (XPS) technique to study chemical properties of the films’ surface and to determine the in-depth compositional profile of the films before and after annealing of an implanted sample. Spectroscopic ellipsometry was used to extract the dielectric function of CuO thin films. Depolarization measurements are shown as a useful method to quickly study differences between similar samples. XPS measurements proved that before annealing there is a peak of Cr concentration in depth of the sample, which is no longer present after annealing. Measurement of film resistance as function of temperature in range of 150–300 °C resulted with 0.82 eV bandgap. Electronic structure obtained with density functional theory calculations (DFT) showed that with Cr doping the energy band gap narrows and the material should become metallic.

## Introduction

Copper oxides are p-type semiconducting materials, that were the first material to show photosensitive properties discovered in 1916 by A.H. Pfund^1^. They can be used in various applications such as sensors, photocatalysis, photovoltaics and antibacterial materials^2–8^. Thin film form of copper oxides is especially favorable as it can be applied as functional layer on desired substrate. Thin film technology uses small amounts of source materials what results in light product, what can be of high importance in photovoltaic applications for space technologies or modules prepared on elastic substrates^9^.

The most used among copper oxides are cuprous oxide Cu_2_O and cupric oxide CuO, the second one being more stable in air ambience and room temperature^10,11^. Moreover, its bandgap value is potentially more beneficial in photovoltaic applications. The bandgap value is reported between 1 and 1.9 eV. This includes the theoretically optimal band gap value for photovoltaic energy conversion as according to Shockley-Queisser theory which is 1.4 eV^12,13^. However, the achieved performance is very poor, and the material requires improvement in the quality of deposited films as well as in its optoelectronic properties. The latter problem can be solved by doping which can be beneficial in photovoltaics and other applications as well^14^. Gezgin et al. using sol-gel method deposited thin films of CuO doped with Zr on silicon to create CuO/Si heterojunctions^15^. Doping at 1% weight level was beneficial for the electrical properties of CuO film and the performance of photovoltaic device, where using non-doped CuO cell showed 0.042% efficiency and for 1% Zr doped CuO efficiency of 0.88%. Saumya et al. showed that Ca doping of CuO nanoparticles improved their antibacterial activity^16^. Doping with Cl or Ce can help to make catalysis of water pollutants degradation more effective^17,18^. Increased absorption of CuO can be beneficial for photocatalytic and photovoltaic applications, using Al as a dopant caused increased absorption of CuO nanoparticles prepared by sol-gel method by Kumar et al.^20^. Ni doping has similar effect as shown by Baturay et al.^19^. Using Mn as dopant improved sensing of isopropanol by nanoflower CuO spheres in study of Chao et al.^21^. Cr was used with success as a dopant in sensing applications, Cr caused electronic sensitisation of CuO nanorods and acted as a catalyst improving sensor selectivity towards NO_2_^22^. This element can improve sensing of various nanostructures of CuO towards ammonia, acetone and H_2_^2,23,24^. Baturay et al. studied the electrical and optical properties of Cr-doped CuO thin films. Using spin coating and annealing at 500°C, they synthesized and characterised films on soda-lime glass and p-type Si (100) substrates. UV–Vis spectroscopy revealed that Cr doping increases transmittance (up to 33% with 3% Cr) and the energy band gap (1.67–2.03 eV). Electrical measurements showed that the 1% Cr-doped CuO/p-Si diode had the best rectification ratio and light sensitivity, highlighting the potential of 3% Cr-doped CuO for photovoltaic applications.

In the work^23^ the electronic structure of CuO doped at the level of 1 Cr atom per 70 Cu atoms was theoretically calculated using the VASP environment. It was shown that at this doping we deal with an isolating gap. Our goal in the work^25^ and the present one was to show the trends in changes of the electronic structure with increasing Cr doping to the CuO insulator. In our case we investigated the region of dopants at the level of one Cr atom per 15 Cu atoms and one Cr atom per 7 Cu atoms. For this purpose, we used one of the most popular and accurate DFT^26^ implemented in the Wien2k^27^ package.

As a continuation of our previous works^25,28,29^, here we use X-ray Photoelectron Spectroscopy (XPS) to analyse surface properties and the in-depth Cu and Cr concentration in CuO thin films implanted with Cr ions and subsequently annealed. Spectroscopic ellipsometry (SE) was used to study dielectric function of the films and their depolarization.

## Methods

The surface chemical properties of CuO thin films were studied with XPS method with an analysis chamber equipped with a 150 mm hemispherical electron energy analyser (Phoibos), which operated in fixed transmission mode with pass energy of 20 eV. Monochromatic Al Kα was the X-ray source with energy of 1486.6 eV at power of 250 W (12.5 kV × 20 mA). Flood gun was used to compensate for the charging effects working at 1 V × 0.1 mA. During data analysis binding energy correction was made with reference to C 1s contamination core level at 286.4 eV. All data analysis and fitting were done with Igor Pro software, and the peaks were fitted using the Voigt function, where all components had the same Lorentzian and Gaussian broadening when coming from the same element, with exception of Cu 2p—here 1/2 and 3/2 peaks had different Lorentzian broadenings. The background functions used for Cu 2p spectra was Total Sum and for other elements the Shirley function. The depth profiling analysis was performed by Ar^+^ sputtering cycles at 3 keV x 10 mA with 1x10^−5^ mbar Ar^+^ pressure, each followed by an XPS measurement. Spectroscopic ellipsometry measurements were performed with J.A. Woollam M-2000 ellipsometer. All measurements were done at 70° incident angle. The data fitting was performed as described in^28^ and was based on a multilayer model with three layers calculated with four Tauc-Lorentz oscillators and the roughness layer calculated as an effective medium approximation of void and the top layer. Dielectric function values were exported from each fitted model layer. The depolarization data is directly obtained from the measurements and there is no fitting needed.

Copper oxide thin films were deposited using the reactive magnetron sputtering method with following parameters: power and current of discharge—50 W and 80–130 mA, respectively; pressure 1.5 × 10^−2^ mbar; atmosphere in chamber 100% O_2_. The 55 nm film was deposited on silicon and 130 nm film on glass. CuO thin films were implanted with Cr+ ions of 10 kV energy—doses: dose 1 —1×10^14^ cm^−2^, dose 2—5×10^14^ cm^−2^, dose 3—1×10^15^ cm^−2^, and 15 kV energy—dose 5x10^16^ cm^−2^. The deposition of films and the implantation experiment are described in^30^. The cited publication shows also X-ray diffraction results that confirm the deposition of CuO monoclinic phase. For XPS depth analysis, a sample with a thickness of 55 nm deposited on Si, was used. It was implanted with 15 keV energy ions, and then half of it was annealed. Ellipsometry measurements were done for thin film samples deposited on glass, of a thickness about 130 nm, the samples were implanted with ions of 10 keV energy. Annealing of samples was done in air at 400 °C for 6 hours. In present work we have carried out calculations with full potential WIEN2K code, in the Generalized Gradient Approximation^26^. The WIEN2K software^27^ as utilized for calculations of electron structure in the ground state (at 0 K). It allows to determine the density of states (DOS), energy gap and electromagnetic radiation absorption presented in^25^. Also, in paper^25^ details of the present calculations are presented. We now deal with the results of the calculations of the dispersion relations for the electronic structure.

The resistance-temperature relationship was measured in a Fine Instruments tube furnace. The temperature was measured with a K-type thermocouple using a Keithley 740 meter. The resistance was measured using the four-contact method using a Keithley 196. Kanthal wires were glued to Au-sputtered contacts on the surface of the tested layer with Dupont 6838 high-temperature conductive silver paste.

## Results and discussion

XPS extracts the signal only from the surface of the sample. Only electrons that escape the surface without undergoing inelastic collisions contribute to the characteristic photoelectron peaks used in XPS analysis. Usually the information is obtained from maximum 10 nm in depth and it depends on the element that is measured. For quantitative analysis there are methods that use atomic sensitivity factors, which are empirically derived factors from known compounds measurement that allow to normalize the peak intensity and then determine the atomic concentration.

**Fig. 1:**
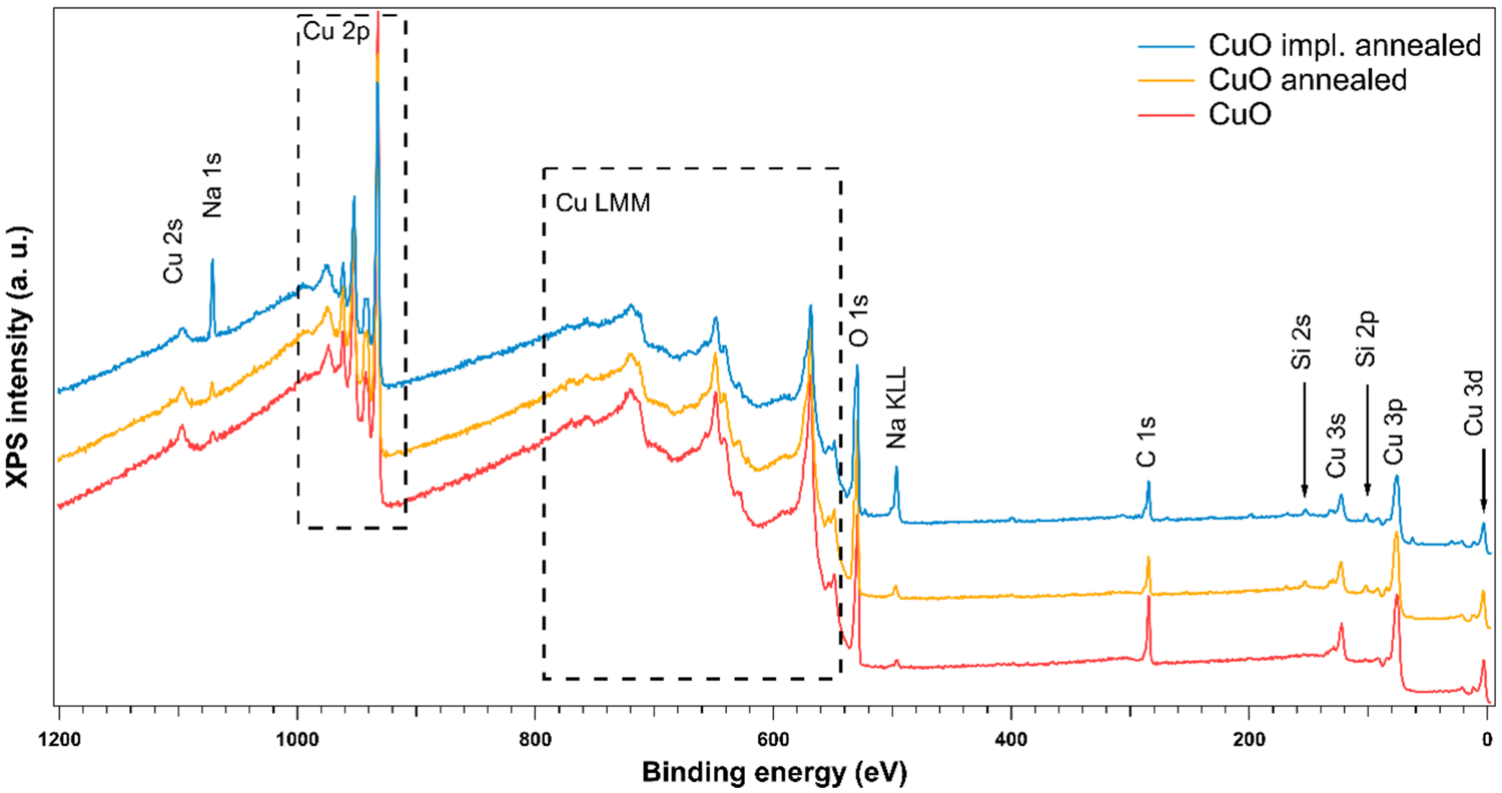
Wide XPS scans of CuO sample: as deposited, annealed, and implanted with 10 keV energy ions and then annealed.

Figure 1 shows wide scans of CuO samples, namely samples (i) as deposited, (ii) after annealing, (iii) after implantation (energy 10 keV, dose 1×10^15^ cm^−2^) and annealing. Wide scans are performed to see what elements are present in the sample. Next, specific ranges are measured to study desired elements’ signal, here Cu 2p, O 1s, Si 2p and Cr 2p. In studied samples clearly copper signals are present, both from photoelectron and Auger peaks. There are some Na and Si detected that could come from the substrate or, more probably, are surface contamination. The Na signal is much stronger in the case of implanted sample.

**Fig. 2:**
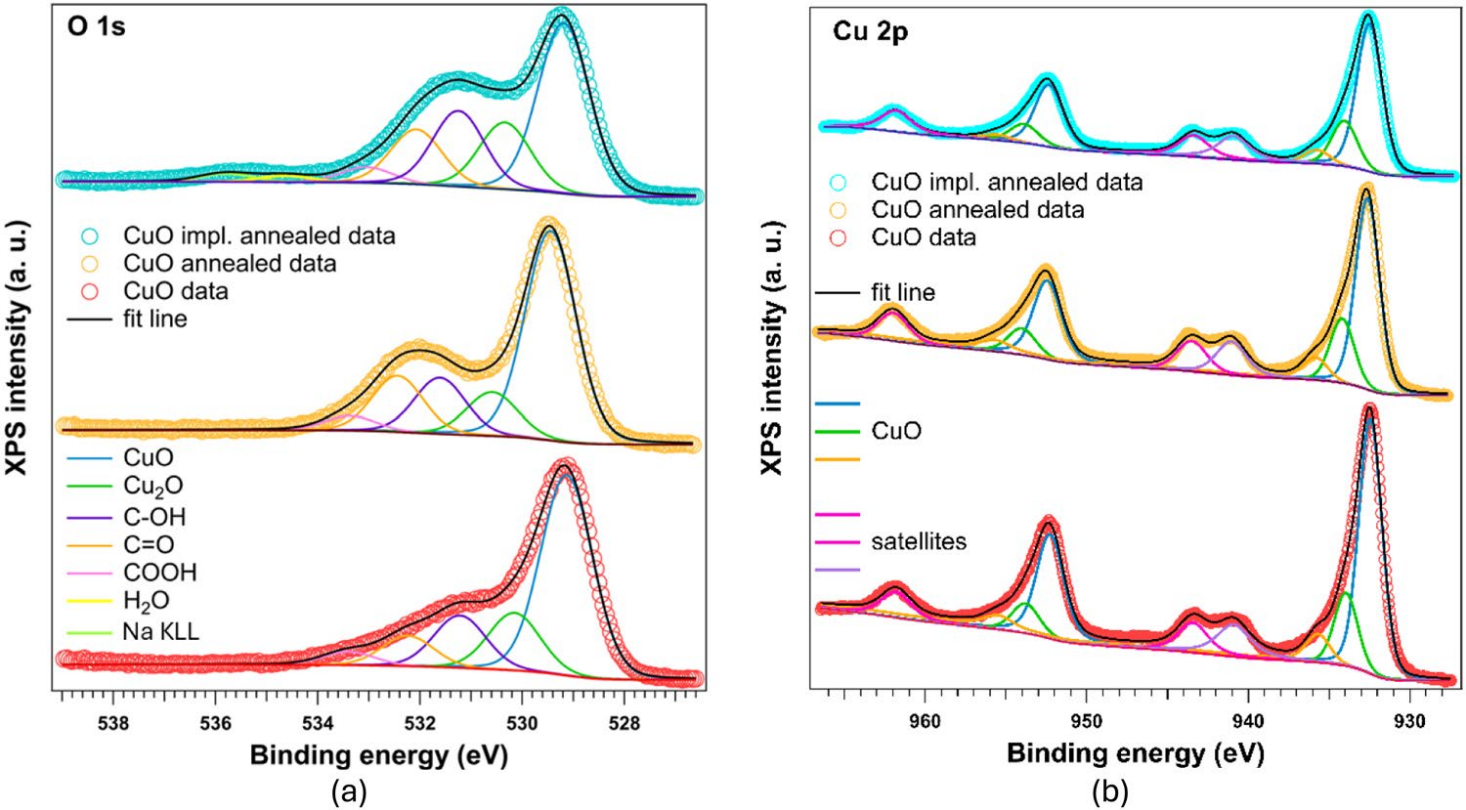
XPS narrow scans for CuO samples at (**a**) O 1s and (**b**) Cu 2p signal. Components in O 1s data are assigned to different origin, while for Cu 2p there is only assignment to CuO lattice or satellite peaks.

The Cu 2p spectra all show typical signal of Cu^2+^ with around 19 eV spin orbit splitting and shake up satellites from the Cu 2p 3/2 peak present at around 9 eV (Fig. 2b)^31^. The most intensive peak comes from CuO lattice and the two weaker from the surface and defects. The as deposited sample has the highest contribution of the lattice component. Figure 2a presents the O 1s spectra which are dominated by a strong peak around 529 eV which corresponds to CuO. Peak at around 530 eV is typically coming from Cu_2_O^32^. The contributions that come from various organic adsorbates are stronger in case of annealed sample. The peak present at around 536 eV is coming from the Na KL_1_L_2,3_ Auger transition^33^. Both annealing and implantation combined with annealing introduce more defects and adsorbates into CuO surface.

**Fig. 3:**
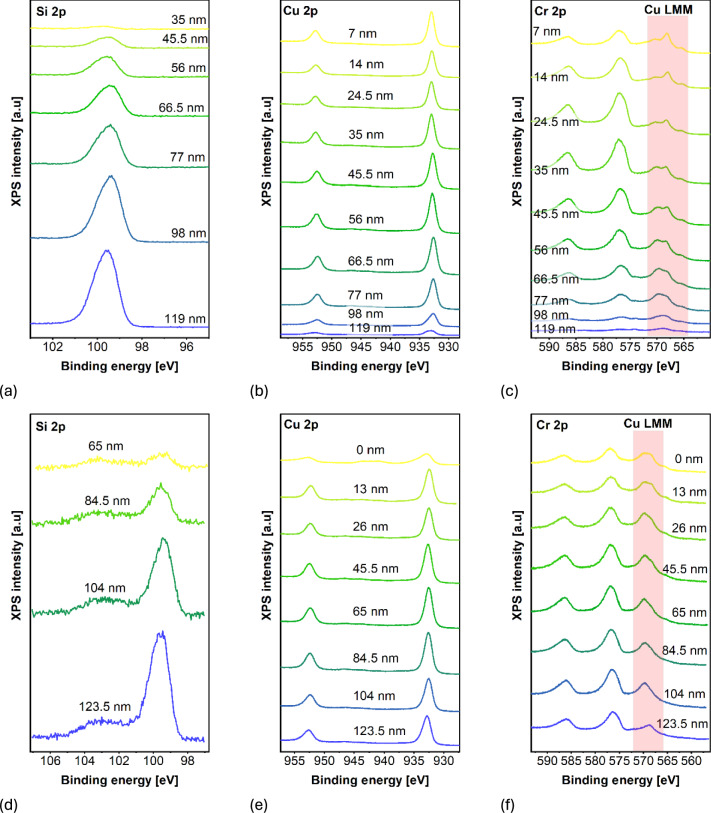
XPS signal in depth of the sample after implantation for (**a**) Si 2p, (**b**) Cu 2p, (**c**) Cr 2p and for sample after implantation and annealing for (**d**) Si 2p, (**e**) Cu 2p, (**f**) Cr 2p.

The depth measurements were performed for CuO deposited on Si with thickness of 55 nm (this is the thickness aimed during deposition process). Thin film was implanted with 15 keV energy Cr ions and dose of 5×10^16^ cm^−2^. These samples were chosen as they were implanted with high dose and therefore were promising to detect Cr signal in them. Half of this sample was then annealing in air at 400 °C for 6 hours. These two samples, implanted, and implanted and annealed, were due to XPS measurements in cycles after Ar sputtering. The resulting spectra are presented in Fig. 3.

The signal from Cr 2p is close to the Cu Auger LMM peaks, which are marked on Fig. 3c,f with light red colour. Using measured data and atomic sensitivity factors (determined for height of peaks^32^) the relative concentration of elements was calculated as in the following equation:

$${A}_{cal}=\frac{A}{ASF}\times corr.$$

Where *A*_*cal*_ is the calibrated amplitude value, *A* is the height of a peak, *ASF* is atomic sensitivity factor and *corr.* is a correction value of maximal amplitude integral. The calculated *A*_*cal*_ is then compared for each element to get the relative concentration.

**Fig. 4:**
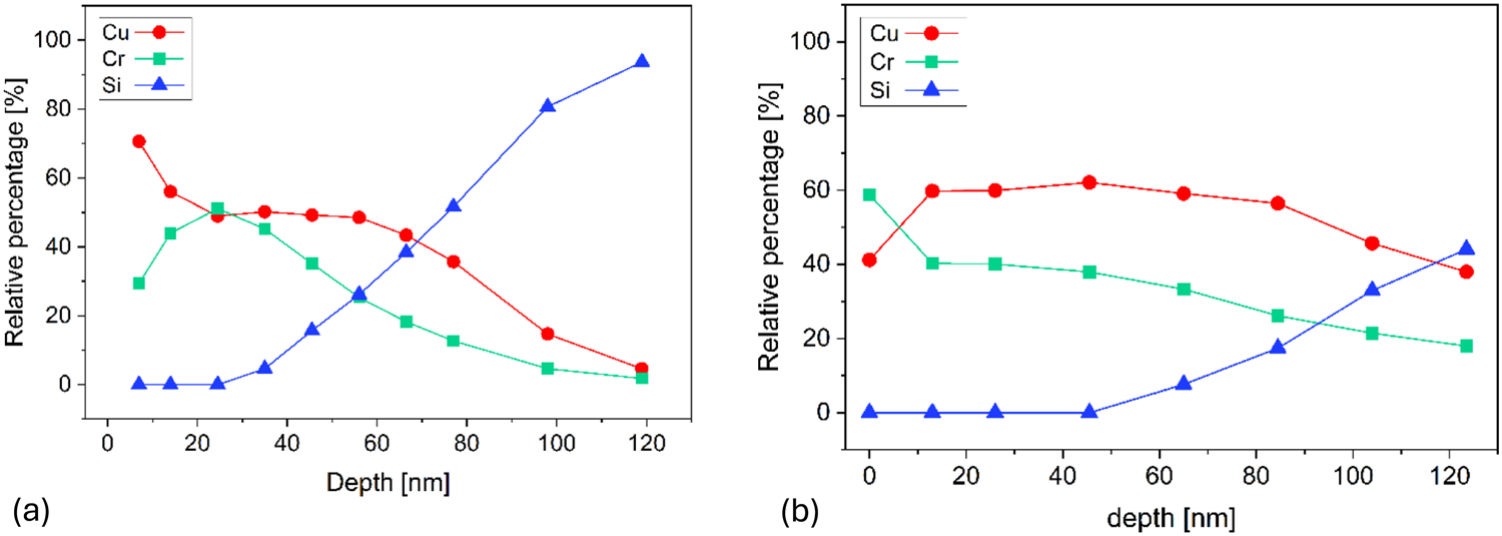
Relative percentage of Cu, Cr and Si in depth of sample (**a**) after implantation, (**b**) after implantation and annealing.

The results are presented in Fig. 4, where the scale of depth does not correspond to the thickness of the films. The depth of measurement was estimated using sputtering rate of the sample, that was determined during sputtering cycles. This calculation is not accurate, yet there is still a very valuable observation to be made. The distribution of Cr before annealing has a clear peak between 20 and 30 nm in depth. After implantation the Cr distribution is almost uniform in the whole sample. This is an expected result from annealing which accelerates diffusion of Cr in CuO. The annealing step distributes the dopant into the film, proving that implantation and annealing can be a successful doping technique for CuO thin films. If the film was thicker, it is probable that the ions would not distribute into the whole thickness of the films. Optimisation of implantation energy, temperature and time of annealing could result with successful doping of thicker samples or manufacturing a doped region within a film.

**Fig. 5:**
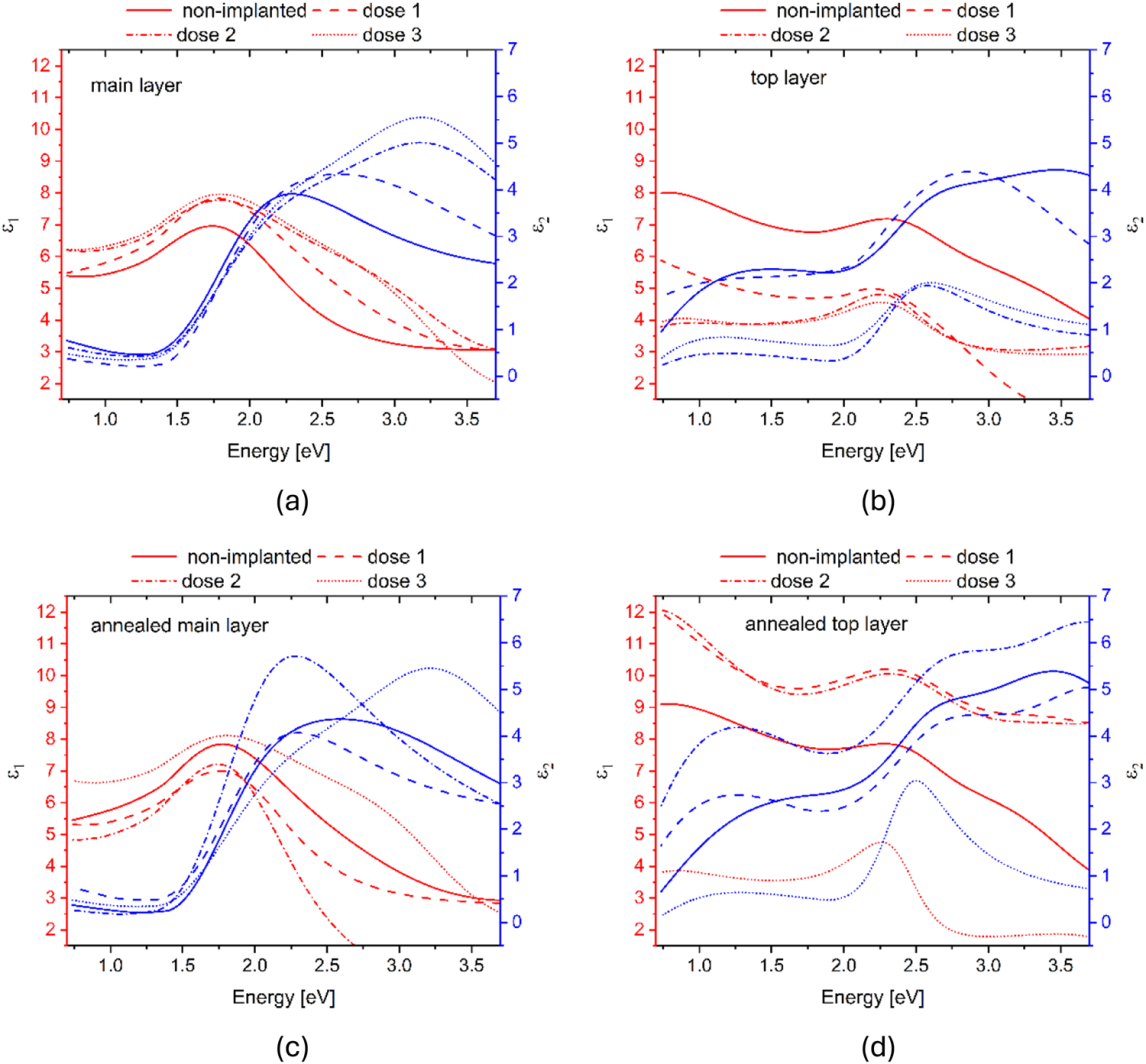
Real and imaginary parts of dielectric function of CuO thin films implanted with 10 keV energy and different doses, data for (**a**) main layer and (**b**) top layer of dielectric model before annealing, data for main (**c**) and top (**d**) layer of model for samples after annealing.

Optical properties analysis was done by fitting of spectroscopic ellipsometry data. Two aspects are showed here, the depolarisation of sample which gives information about the uniformity of the film. From modelling with models described in^28^ there is the real and imaginary parts of dielectric function presented for the main middle layer in the model and for the top layer in the model in Fig. 5. The most important observation is that the top layer dielectric function changed much more than the main layer, which is the region where the implantation has the most influence on material properties.

**Fig. 6:**
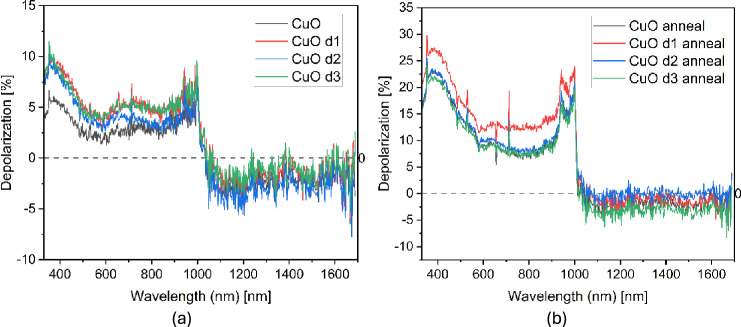
Depolarisation parameter for CuO samples (**a**) before and (**b**) after annealing, d1, d2, d3 correspond to dose 1, dose 2, dose 3 of Cr ion implantation.

The ideal depolarization of a sample is 0, but the samples are not perfect materials. Depolarization measured with spectroscopic ellipsometer from samples before and after implantation and/or annealing are presented in Fig. 6. The most important observation here is that the sample dose 1 after annealing differs the most from the non-implanted annealed sample, among all the samples. This indicates that the optical and physical properties of this sample are different the most. Depolarization of a sample depends on any backside reflection that can reach the detector and on the uniformity of the sample below over the measured area and in depth of the film.

**Fig. 7:**
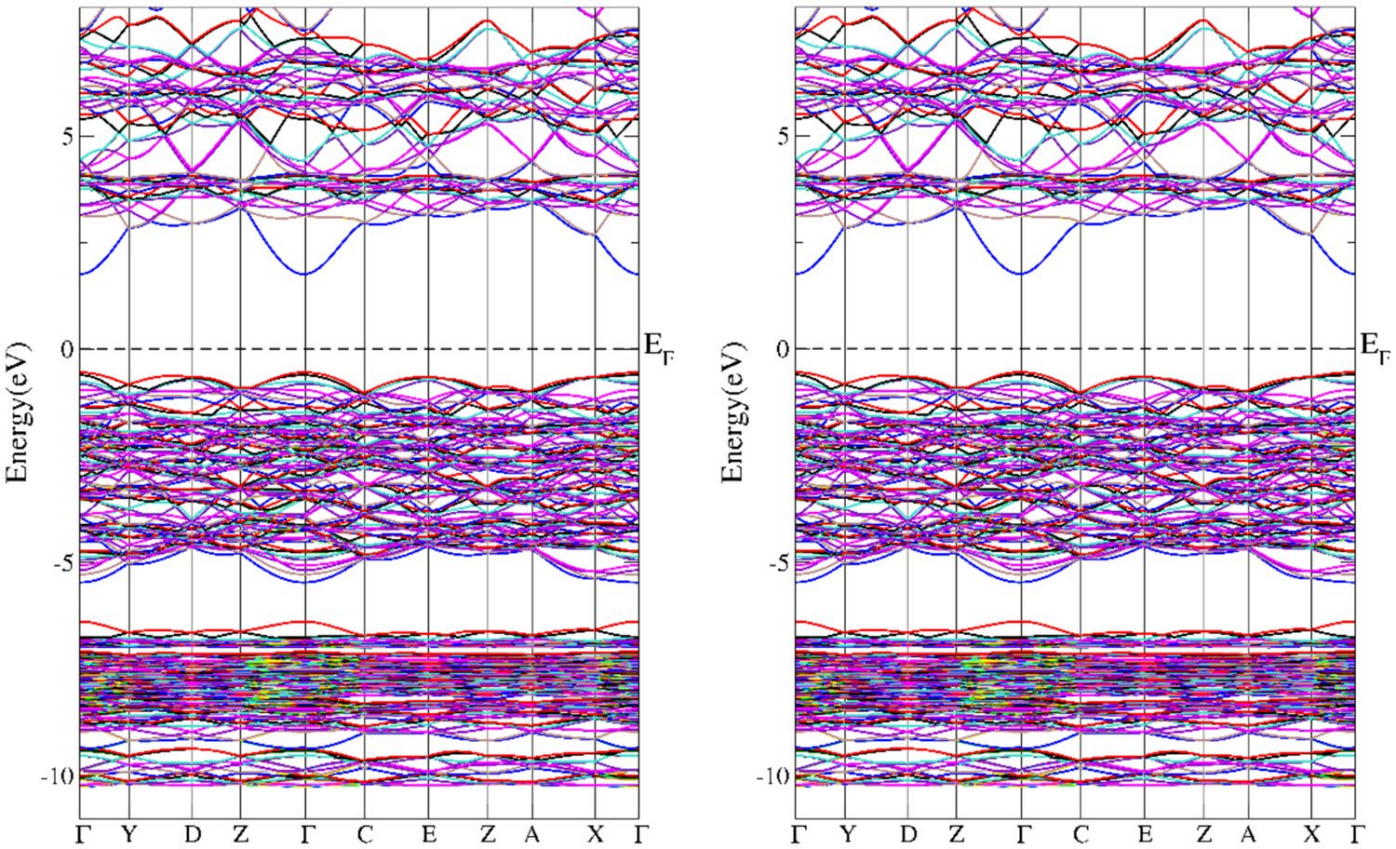
Calculated band structure for the CuO, left spun up, right spin down.

**Fig. 8:**
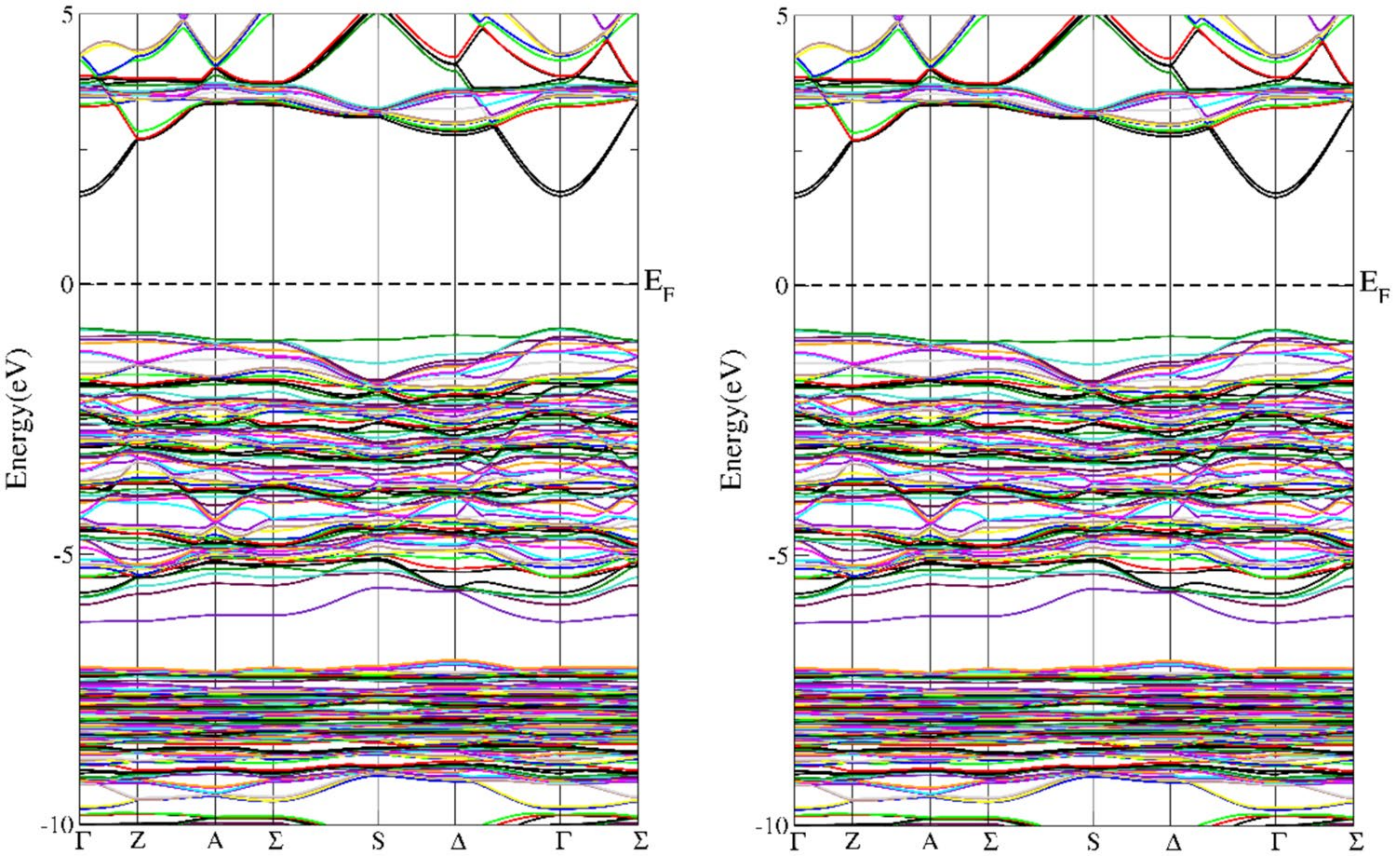
Calculated band structure for the Cu_15_CrO_16_. Spin up left side and spin down right side.

**Fig. 9:**
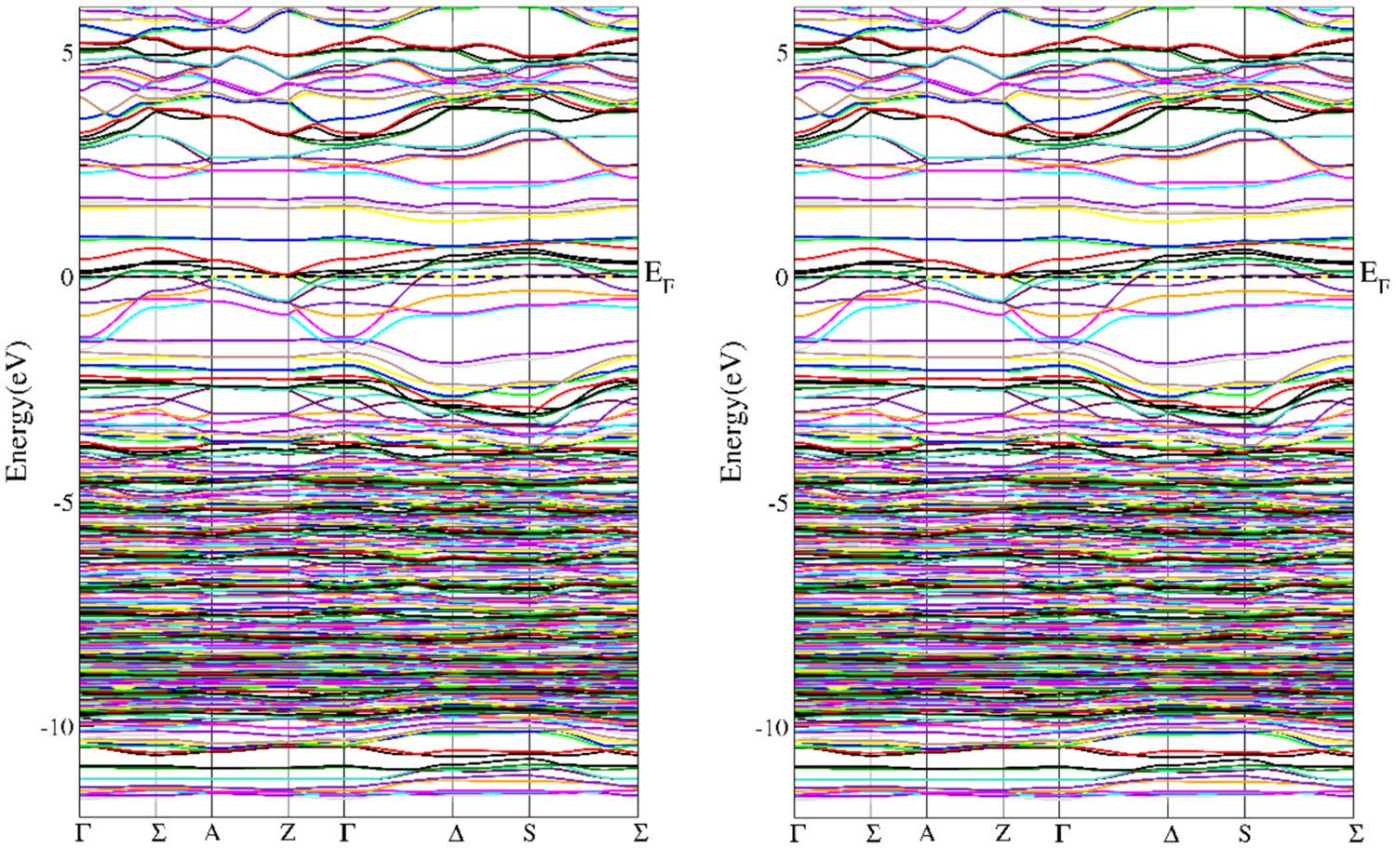
Calculated band structure for the Cu_7_CrO_8_. Spin up left side and spin down right side.

In addition to the information on the electronic structure, it is important to extend the knowledge of the electronic structure obtained in XPS measurements with details in the fermi energy environment. In particular, it is necessary to check whether in the tested Cr-implanted materials we are dealing with a change in the nature of the material from insulating to conducting as the Cr concentration increases. For this purpose, theoretical calculations of the electronic structure were carried out. It was found that in the case of doping at the level of one Cr atom per 14 copper atoms, a semi-metallic character visible in DOS was observed for the ideal structure^9^. In the band structure shown in the Figs. 7, 8 in the presented directions of the reciprocal lattice, we can see that this structure for both CuO and Cu_15_CrO_15_ looks similar and has a similar energy gap. The situation changes radically in the case of doping at the level of one Cr atom per 7 Cu atoms, then the material theoretically has a conducting character with electronic states at the fermi level (see Fig. 9).

**Fig. 10:**
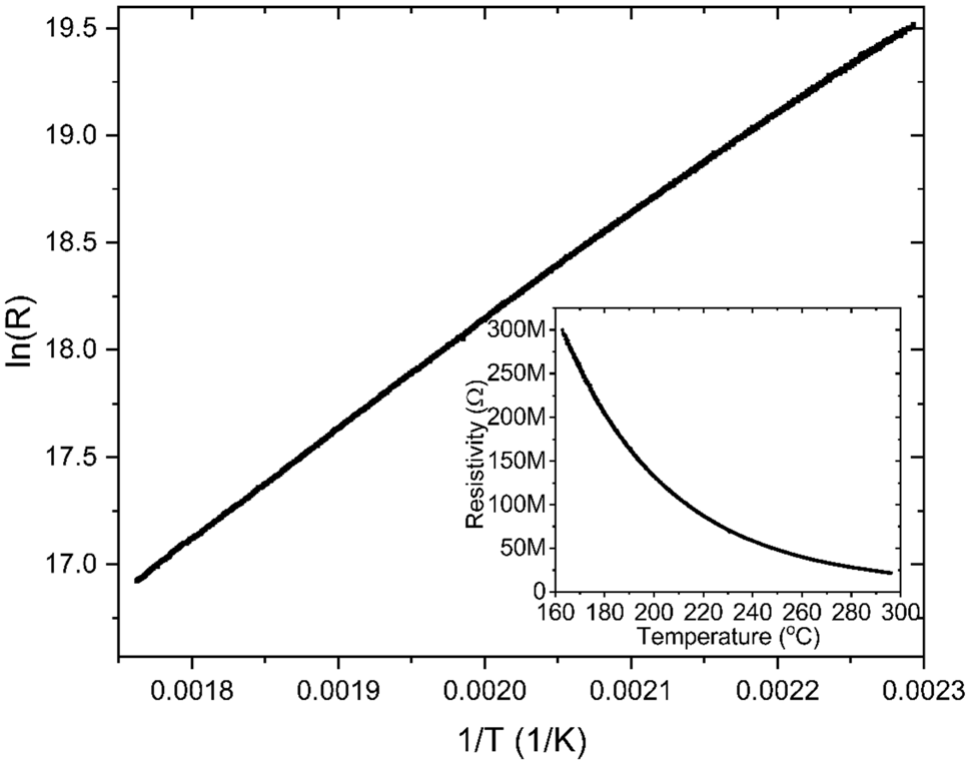
Logarithm of measured resistivity as a function of inverse temperature for CuO thin layer with Cr implanted. On insert dependence of resistivity versus temperature.

To check what the situation is like in the case of a real sample obtained as a result of bombardment with accelerated Cr ions, the dependence of resistance on temperature was measured. As a result, a classical dependence for semiconductors was obtained, presented as in the Fig. 10 insert. In order to determine the energy gap, the dependence of ln(R) on 1/T was plotted, where R is resistance and T is temperature. In such a plotted dependence, the slope coefficient of the straight line is equal to ΔE/2k_B_. ∆E is the width of the energy gap and k_B_ is the Boltzman constant. The width of the energy gap determined on this basis was ∆E = 0.82 eV.

## Conclusions

XPS was utilised to create a depth concentration profile of Cr in CuO thin films. The measurements were successful, clearly showing a peak in Cr concentration for implanted CuO sample. After annealing, Cr distribution became uniform throughout the film. Dielectric function of samples was modelled and studied with spectroscopic ellipsometry. The characteristics of both real and imaginary parts of dielectric function changed the most for the top layer of the model, which is the part of material mostly influenced by the implantation process. Observation of depolarization is useful to give qualitative results and insights, which showed that CuO film implanted with dose of 1x10^14^ cm^-2^ and energy 10 keV of Cr ions differed the most after annealing in reference to annealed non implanted sample.

The results of band structure calculations suggest that with increasing Cr doping the material changes from an insulating state in CuO to a semimetal and finally to a metallic state at the doping level of one Cr atom per 7 Cu atoms. In the material obtained as a result of implantation with Cr+ ions a semiconductor structure with a gap of 0.82 eV was obtained with resistance measurement. The difference between the modeled electronic structure and the actual one may result from the fact that the implantation process may change the local crystal structure in the Cr ion transition path and in its proximity due to recoil cascades. Thanks to this preparation method at a higher Cr concentration we can still have an energy gap.

## Data Availability

The data of presented results is available from the corresponding author upon reasonable request.
